# Peripheral Transplantation of Mesenchymal Stem Cells at Sepsis Convalescence Improves Cognitive Function of Sepsis Surviving Mice

**DOI:** 10.1155/2022/6897765

**Published:** 2022-09-19

**Authors:** Longjiao Tan, Yong Cheng, Hui Wang, Jianbin Tong, Xian Qin

**Affiliations:** ^1^Hunan Province Key Laboratory of Brain Homeostasis, Third Xiangya Hospital, Central South University, Changsha, 410013 Hunan, China; ^2^Department of Anatomy and Neurobiology, School of Basic Medical Sciences, Central South University, Changsha, 410013 Hunan, China; ^3^Department of Plastic and Burns Surgery, Huazhong University of Science and Technology Union Shenzhen Hospital, Shenzhen, Guangdong, China; ^4^Department of Anesthesiology, Third Xiangya Hospital, Central South University, Changsha, 410013 Hunan, China

## Abstract

**Objective:**

To investigate the effects of peripheral transplantation of mesenchymal stem cells (MSCs) at sepsis convalescence on post-sepsis cognitive function and underlying mechanisms in mice.

**Methods:**

Sepsis was induced by cecal ligation and puncture (CLP) in mice. Bone marrow-derived MSCs from mice were cultured and injected via tail vein on the 8^th^ day after CLP. Cognitive function was detected in open field, novel object recognition task, and delayed matching-to-place water maze task during 10-26 days after CLP. Neuroinflammation, neurogenesis, and peripheral inflammation were detected on the 12^th^ and 31^th^ days after CLP. MSCs tracing was detected during 8-10 days after CLP.

**Results:**

Transplanted MSCs were located at peripheral organs (lung, spleen, liver) and had no obvious effects on survival and weight of sepsis mice. Transplanted MSCs mitigated cognitive impairments and hippocampal microglial activation, improved hippocampal neurogenesis of sepsis surviving mice, and had no obvious effect on the leukocyte amount, the neutrophil percentage, and the inflammatory factors of peripheral blood, and the hippocampal inflammatory factors.

**Conclusions:**

Our data indicated that MSCs transplantation via peripheral vein at later phase of sepsis can improve post-sepsis cognitive impairment and hippocampal neurogenesis of sepsis surviving mice.

## 1. Introduction

Sepsis and its associated complications are one of the foremost causations of death in intensive care units worldwide [1, 2]. More than half of septic patients have sepsis-associated encephalopathy (SAE), which remains badly understood and is deemed to be reversible [2, 3]. However, septic survivors who had been diagnosed with SAE may have permanent neurocognitive dysfunction, which could result in some serious socioeconomic burden [3–5]. Currently, SAE is mainly considered to be a peripheral inflammation-induced brain dysfunction [3]. However, numerous efforts focusing on targeting the inflammatory cytokines network have proved unprofitable for the treatments of sepsis and SAE [6]. The search for novel potential therapeutic targets still needs to be continued.

Stem cell therapy is proved to be beneficial for central nervous system diseases, such as Alzheimer's diseases, Parkinson's diseases, and traumatic brain injury, due to the self-renewal potentialities, multiple differentiation activities, neurotrophic properties, and immuno-regulatory effects of the stem cells [7, 8]. A recent study has demonstrated that bone marrow-derived mesenchymal stem cell (BM-MSCs) transplantation in the first 6 hours after sepsis improves the cognitive and behavioral impairments of septic surviving mice [9]. However, if the MSCs treatment will be applied to the sepsis patients at the early phase to prevent cognitive and behavioral alterations, some problems should be taken into account. First, symptomatic support treatment and anti-inflammatory treatment are preferred at the early phase of sepsis patients. Second, complicated inflammatory environment is a main feature of sepsis at the early phase [10], which can affect the efficacy of MSCs treatment [11]. In contrast, it may be easy to implement and achieve stable therapeutic effects, if the stem cell therapy is given during sepsis convalescence.

The present study investigated whether peripheral administration of MSCs at the sepsis convalescence could mitigates post-sepsis cognitive function in the well-established cecal ligation and puncture (CLP) model mice of sepsis. Toward this end, we further explored the underlying mechanisms of MSCs treatment.

## 2. Materials and Methods

### 2.1. Animals

Adult old C57BL/6 J male mice weighing 25-30 g were obtained from the experimental animal center of Central South University, China. The protocol [LLSC(LA)2017-061] was approved by the ethics committee of the 3rd Xiangya Hospital of Central South University. All mice were housed under a 12 h light-dark cycle, an adequate temperature of 23°C, and a relative humidity of 50%-60% with free access to water and food. Animals were utilized with age- and weight-match and also were treated to minimize their suffering in experiments.

### 2.2. Sepsis Model

Mice were randomly divided into Sham+NS, Sham+MSC, CLP + NS, and CLP + MSC groups. The last two groups received CLP surgery, while the former two groups did not. CLP surgery was performed as previously described [12, 13]. Briefly, animals were anesthetized with the 2% sevoflurane (Maruishi Pharmaceutical Co., Ltd., Japan) in a well-ventilated room. 40% of the cecum was ligated and punctured once with a 20-gauge needle. The Sham+NS and Sham+MSC groups underwent only identical laparotomy but without CLP. Bupivacaine (3 mg/kg) and Buprenorphine (0.1 mg/kg) were injected subcutaneously once to avoid postoperative pain. All animals were given 1 mL of saline subcutaneously every 8 hours for 5 days.

### 2.3. MSC Culture and Delivery

Bone marrow-derived MSCs (Cyagen Biosciences Inc, Guangzhou, China) from the C57BL/6 J mice were cultured in an incubator (Thermo, USA) under an appropriately maintained temperature of 37°C and an atmosphere of 5% CO_2_. Culture medium (MUBMX-90011, Cyagen) was subsequently renewed every 2 days after removing nonadherent cells on day 2. When the cells population density reached 80%-90%, it was digested by 0.25% trypsin and subcultured according to the density of 2.5 ~ 4.0 × 10^4^/cm^2^. The mice in CLP + MSC and Sham+MSC groups were injected with MSCs (1 × 10^6^ diluted in 200 *μ*l of physiological saline) within twelve passages via tail vein on the 8^th^ day after CLP surgery, while the other groups were admitted equal volume of normal saline.

### 2.4. MSC Labeling and Tracing

The MSCs were directly labeled via incubating in 1 *μ*M Lipophilic Tracers DiR (Invitrogen, USA) for 30 minutes at 37°C [14] . MSCs then were washed twice with new culture medium for 10 minutes each time to remove residual DiR solution. Mice were injected with 3 × 10^6^ of DiR labeled MSCs (1 × 10^6^ diluted in 200 *μ*l of normal saline) via tail vein on the 8^th^ day after surgery. These mice were sacrificed at 6 h, 24 h, or 72 h after injection, and the organs (brain, lung, kidney, spleen, liver) were dissected for fluorescent signal by IVIS Lumina II (PerkinElmer, USA). The fluorescence intensity was quantified with ImageJ in a blind manner.

### 2.5. Behavior Test

**Figure 1:**
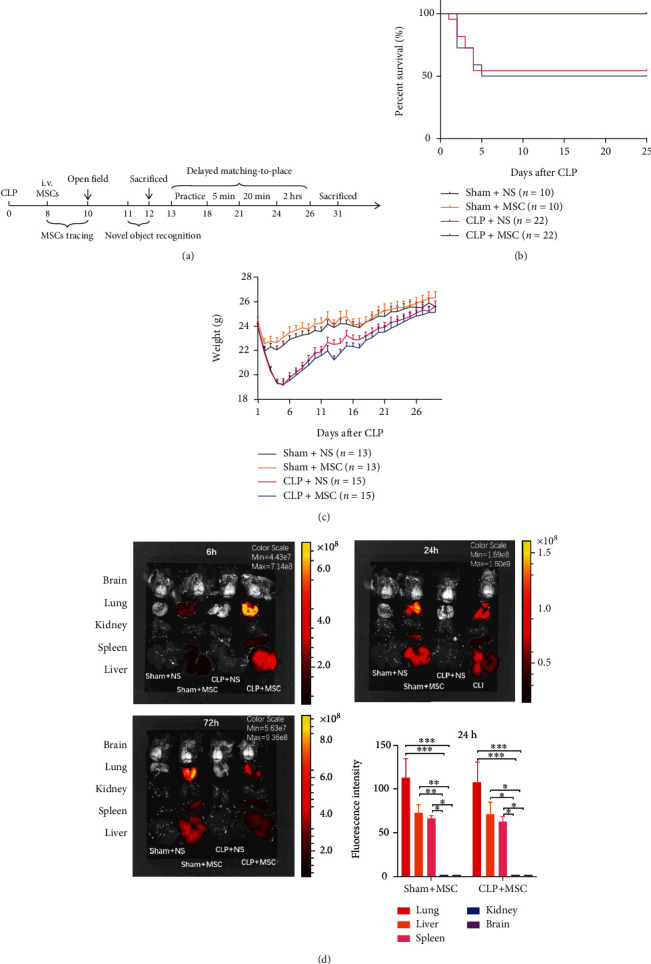
Transplanted MSCs were located at peripheral organs and had no obvious side effects. (a) Experimental time course for CLP, MSCs transplantation, and behavioral tests. (b) The survival of each group during 0-25 days after CLP surgery (Sham+NS *N* = 10, Sham+MSC *N* = 10, CLP + NS *N* = 22, CLP + MSC *N* = 22; Log-rank (Mantel-Cox) test, *p* < 0.05); (c) The body weight recovery of each group during experimental phases (Sham+NS *N* = 13, Sham+MSC *N* = 13, CLP + NS *N* = 15, CLP + MSC *N* = 15; two-way ANOVA followed by multiple comparison tests); (d) Fluorescent signal was detected in peripheral organs (lung, spleen, liver) at 6 h, 24 h, and 72 h after CLP surgery (*N* = 3 for each group/time point). Sham+NS: mice received laparotomy and tail vein injection of normal saline; Sham+MSC: mice received laparotomy and tail vein injection of MSCs; CLP + NS: mice received CLP surgery and tail vein injection of normal saline CLP + MSC: mice received CLP surgery and tail vein injection of MSCs.

Mice were subjected to open field, novel object recognition, and delayed matching-to-place tasks on postoperative day 10-26 as shown in Figure 1(a). Before the test began, mice were placed in a sound-isolated behavioral testing room for half an hour. All tests were carried out by the same person who was blind to the animal's group.

#### 2.5.1. Open Field Test

Open field test was used to assess locomotive activities. Each mouse was gently placed in the center of apparatus (width:50 cm, length:50 cm, height:38 cm) and was left alone to explore the arena for 5 min. At the end, the mouse was immediately taken back to its home cage. Using 75% alcohol solution to clean the apparatus after the fecal and urine were removed left by the previous mouse. The total ambulatory distances and time in central zone were analyzed.

#### 2.5.2. Novel Object Recognition (NOR) Test

Object recognition experiment was conducted to test hippocampus-related learning and memory described in previously studies [15]. It was performed in a 30 cm ×30 cm ×38 cm open-field apparatus. The test included training and testing phases. In the training phase, two identical objects were parallelized equidistant from the center of arena, and equidistant from the arena walls. The mice were gently placed in the arena keeping their heads away from the two identical objects. Then, the mice were permitted to freely explore the objects for 10 min. 24 hours later, one of the familiar objects was replaced with a novel object, and animals were also allowed to freely explore for 10 min. The apparatus and objects were cleaned with 75% alcohol solution between trials to remove the remaining scent. The behaviors were recorded using a digital camera. The preference index (defined as novel object investigation time/(novel object investigation time + familiar object investigation time)) was used to assess the learning and memory.

#### 2.5.3. Delayed Matching-to-Place (DMP) Task

The DMP water maze task is used to evaluate working memory [16–18]. Following the reported protocol [16–18], mice were pretrained using four trials per day for 5 days. After pre-training, the mice were given the testing tasks using memory intervals (intertrial intervals between trials 1 and 2) of 5 sec, 20 min, and 2 h. Each testing phase period was 3 days. Testing phase performance was recorded by an overhead video camera (Logitech, China) connected to video recorder and a computer running custom-written Smart 3.0 software, and was calculated by subtracting the trial 2 time/path-length for each mouse from its trial 1 time/path-length. Greater time/path-length differences indicated better performance.

### 2.6. Immunohistochemisty

Hippocampal neurogenesis and microglia were measured using the doublecortin (DCX) and Iba-1 staining, respectively. On the 12^th^ and 31^th^ days after CLP surgery, mice were deeply anesthetized with inhaled sevoflurane, and were perfused with 0.01 M PBS and 4% PFA. The brains were collected and postfixed in 4% PFA for 24 h at 4°C. After dehydrating in 15% and 30% sucrose at 4°C, brains were cut into 20 *μ*m thick serial sections using a freezing microtome (Leica CM1950, Eetzlar, Germany). Sections of brains were treated with 3% hydrogen peroxide (H2O2) for 10 min, were blocked with 5% bovine serum albumin (BSA, Sigma, MO, USA) for 1 hour at room temperature, and were incubated in primary antibodies (rabbit anti-Iba1: 1 : 500, Wako, Japan; rabbit anti- DCX (1 : 500, Cell Signaling Technology, Danvers, United states) at 4°C overnight and secondary antibody (Iba-1: goat-anti rabbit, Vector Laboratories; DCX: 1 : 400, Alexa Fluor® 488, abcam, USA) for 2 h at room temperature. For the Iba-1 staining, immunoreaction products were visualized by DAB Kit (Beijing Zhongshan Jinqiao Biological Technology Co., Ltd, China) while the DCX staining slices were cover- slipped in anti-fade mounting medium (VECTASHIELD® with DAPI, Vector labs, USA).

The photographs were acquired under a microscope (Nikon, Tokyo, Japan) and LSM800 confocal microscope (Carl Zeiss, Jane, Germany). Based on the Iba-1 staining (*n* = 12 slices from three mice for each group), the number of Iba-1 positive (Iba-1^+^) microglia and the percent of activated microglia in the CA1 and dentate gyrus (DG) were counted and analyzed as described by our previous study [19]. According to the report, resting microglia was defined as when the cell body was small and round and the branches were thin, highly ramified, and equally distributed around the cell body. In contrast, activated microglia was defined as when the cell body was bigger, pleomorphic bi- or tri-polar, or spindle/rod-shaped, and the branches were shortened, twisted or displayed no ramification [19]. For the DCX staining, according to the reports [20, 21], the number of DCX positive (DCX^+^) cell and DCX^+^ fibers were computed in subgranular zone (SGZ) and middle molecular layer (MLm) of the entire DG (*n* = 9 slices, ×400). Three mice were counted for each group.

### 2.7. Western Blot

The hippocampal tissues were collected on the 12^th^ and 31^th^ days after CLP surgery, and homogenized in cold lysis buffer. The supernatants were collected and their protein concentrations were determined using a BCA protein assay kit (CWbio, China) according to the manufacturer's instructions. Samples were separated by 10% SDS-polyacrylamide gel electrophoresis and transferred to PVDF membranes, which were subsequently blocked using 10% milk for 1 h. Blots were detected using primary antibodies against PSD-95 (1 : 1000, Cell signaling technology, Denver, USA), synaptophysin (1 : 2000, lot 17785-1-AP, proteintech), and *α*-tubulin (1 : 1500, lot 60008-1-lg, proteintech). Subsequently, after three times washed, blots were incubated with a secondary antibody (1 : 8000, 926-32211, LI-COR®, United States). Finally, the proteins were visualized with Odyssey-CLX infrared imaging systems (LI-COR®, Lincoln, United States). Protein levels were quantified by densitometry using Image J software (National Institutes of Health, MD, United States) and were, respectively, normalized to *α*-tubulin.

### 2.8. Quantitative Real-Time PCR (RT-qPCR) Assay

Total RNAs of hippocampal tissues were extracted using the Trizol Reagent (Invitrogen, United States) and reverse transcribed into complementary DNA using a cDNA Synthesis Kit (GeneCopoeia, United States) according to the manufacturer's instructions. RT-qPCR was performed with the mRNA qPCR mix (GeneCopoeia, United States) accordingly. The primers (Table 1) for all assayed genes were determined utilizing revealed sequences as listed. The annealing temperature was 60°C, and the reaction was conducted utilizing LightCycler®480 II analyzer (Roche, Mannheim, Germany).

### 2.9. Peripheral Blood White Blood Cell Counting and ELISA Assay

The blood was collected from the right atrium into EDTA-coated tubes for white blood cell counts and high mobility group box 1 (HMGB1) protein detection on the 12^th^ day after CLP surgery. After a three-fold dilution with saline, the blood was rapidly calculated by a Mindray BC-5300 blood analyzer. HMGB1 concentration of plasma was measured using ELISA (Nanjing Jiancheng Biological Engineering Research Institute, China) according to the manufacturer's instructions. Six mice were detected for each group.

### 2.10. Statistical Analysis

All data were analyzed with Prism 9 (Graphpad Software Inc., La Jolla, CA, USA), and were presented as mean ± standard error (SEM). Log-rank (Mantel-Cox) test was used to analyze the factors of the survival time for any significant differences. The body weight changes and DMP task performance were analyzed with two-way ANOVA followed by multiple comparison tests. The one-way ANOVA followed by multiple comparison tests was used for other results. A significant level was set at *p* < 0.05.

## 3. Results

### 3.1. Transplanted MSCs Were Located at Peripheral Organs and Had no Obvious Side Effects

To determine the safety of MSCs transplantation at later phase of sepsis, we detected the survival and weight of sepsis mice (Figure 1(a). Compared to the Sham+NS and Sham+MSC groups, the survival rate and weight of CLP + NS group decrease significantly (Figures 1(b) and 1(c)). There were no significant difference of survival rate and weight between CLP + NS and CLP + MSC groups (Figures 1(b) and 1(c)). These suggested no obvious side effects of MSCs transplantation at later phase of sepsis. To trace the transplanted MSCs with DiR marker, we detected the fluorescent signal of the organs (brain, lung, kidney, spleen, liver) at 6 h, 24 h, and 72 h after tail vein injection. Fluorescent signal was mainly detected in lung, spleen, and liver. (Figure 1(d), suggesting that MSCs were mainly located at peripheral organs.

### 3.2. MSCs Transplantation Mitigated Cognitive Impairments of Sepsis Surviving Mice

**Figure 2:**
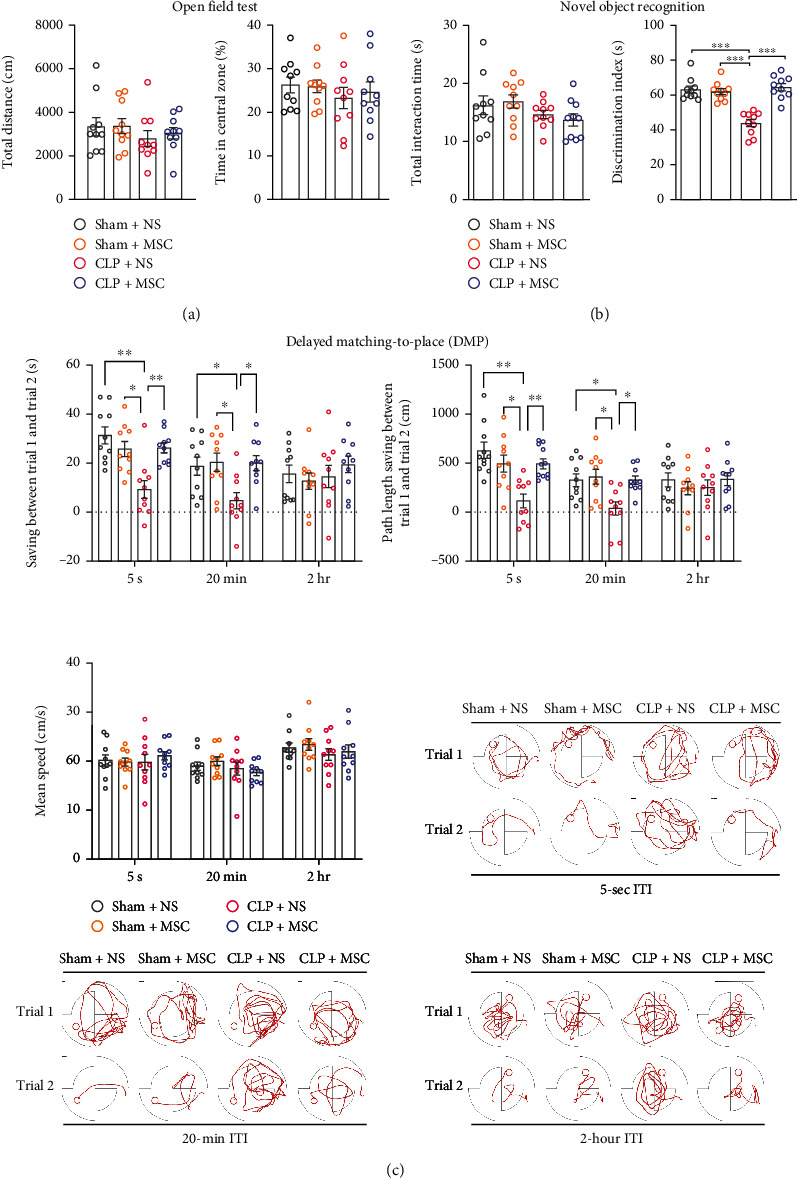
MSCs transplantation mitigated cognitive impairment of sepsis survival mice are as follows: (a) total distance and time in center zone of open field test on the 10^th^ day after CLP surgery (One-way ANOVA followed by multiple comparison tests, *N* = 10 for each group); (b) preference index for novel object in novel object recognition task on the 11^th^ and 12^th^ days after CLP surgery (One-way ANOVA followed by multiple comparison tests, *N* = 10 for each group); and (c) the performances of delayed matching-to-place (DMP) task in a water maze from the 13^th^ to 26^th^ days after CLP surgery. Greater latency and path length saving between trial 1 and trial 2 with intertrial intervals (ITI) of 5 sec, 20 min, or 2 h indicated better performance (two-way ANOVA followed by multiple comparison tests, *N* = 10 for each group). The data were expressed as mean ± SEM; ^∗^*P* < 0 · 05, ^∗∗^*P* < 0 · 01, ^∗∗∗^*P* < 0 · 001.Sham+NS: mice received laparotomy and tail vein injection of normal saline. Sham+MSC: mice received laparotomy and tail vein injection of MSCs; CLP + NS: mice received CLP surgery and tail vein injection of normal saline; CLP + MSC: mice received CLP surgery and tail vein injection of MSCs.

We next investigated whether MSCs transplantation at later phase of sepsis could improve cognitive function of sepsis surviving mice via open field test, novel object recognition test and delayed matching-to-place (DMP) water maze task (Figure 1(a). There was no significant difference between four groups in open field test (Figure 2(a). In the novel object recognition test, the preference index of the CLP + NS group was significantly less than that of Sham+NS, Sham+MSC, and CLP + MSC groups (Figure 2(b). DMP task was used to evaluate working memory which began at 13 days after CLP surgery. In this task, mice were pre-trained for 5 days and then, tested using four sessions with intertrial intervals of 5 sec, 20 min, or 2 h between trials 1 and 2. Greater latency and path-length saving between trial 1 and trial 2 indicated better performance. The latency and path length saving of CLP + NS group at 5 s and 20 min intertrial intervals (ITI) were significantly less than that of Sham+NS, Sham+MSC, and CLP + MSC groups (Figure 2(c). This above information supported an improvement of cognitive function of sepsis surviving mice with MSCs transplantation at later phase of sepsis.

### 3.3. MSCs Transplantation Improved Hippocampal Neurogenesis of Sepsis Surviving Mice

**Figure 3:**
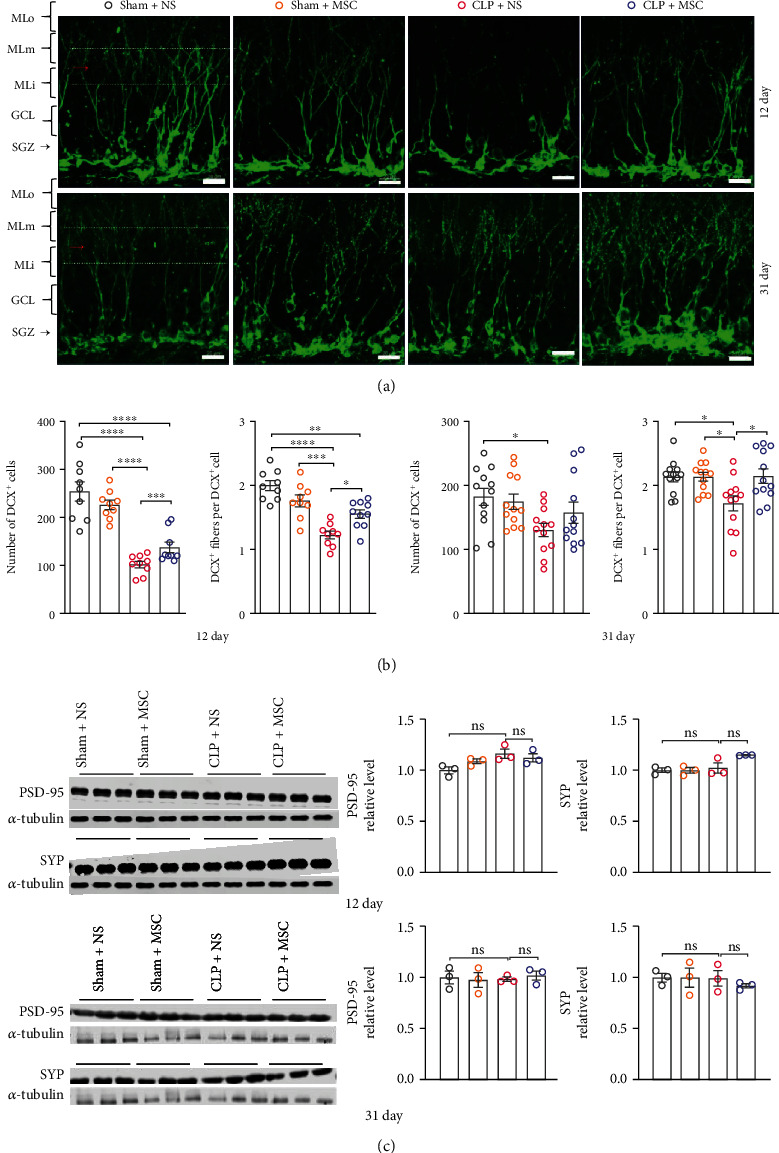
MSCs transplantation improved hippocampal neurogenesis of sepsis survival mice. (a) Representative images of doublecortin (DCX) staining in the hippocampus on the 12th and 31th days after CLP surgery (bar = 20 *μ*m); (b) number of DCX+ cells in dentate gyrus of the hippocampus and the density of DCX+ fibers in middle molecular layer (MLm) on the 12^th^ and 31^th^ days after CLP surgery (unpaired *t* test, *N* = 9 sections from 3 mice per group); (c) representative western blot analysis of PSD-95 and synaptophysin (SYP) in the hippocampus on the 12^th^ and 31^th^ days after CLP surgery (One-way ANOVA followed by multiple comparison tests, *N* = 3 mice per group). The data were expressed as mean ± SEM; ^∗^*P* < 0 · 05, ^∗∗^*P* < 0 · 01, ^∗∗∗^*P* < 0 · 001. Ns: no significance; Molecular layer (ML) of DG was divided into 3 sub-regions of inner (MLi), middle (MLm), and outer (MLo). GCL indicates granule cell layer. SGZ indicates subgranular zone; Sham+NS: mice received laparotomy and tail vein injection of normal saline; Sham+MSC: mice received laparotomy and tail vein injection of MSCs; CLP + NS: mice received CLP surgery and tail vein injection of normal saline; CLP + MSC: mice received CLP surgery and tail vein injection of MSCs.

Neuroplasticity is the basis of cognitive function and is usually evaluated by the adult neurogenesis marker doublecortin (DCX) and synaptic protein postsynaptic density-95 (PSD-95) and synaptophysin in the hippocampus. Comparing with Sham+NS and Sham+MSC groups, the number of DCX^+^ cell and DCX^+^ fibers (middle molecular layer, MLm) per DCX^+^ cell of entire DG on the 12^th^ and 31^th^ days after CLP surgery in CLP + NS group significantly decreased, which were significantly improved in CLP + MSC group (Figures 3(a) and 3(b), and Figure S1). In contrast, no significant changes of hippocampal PSD-95 and synaptophysin were detected between Sham+NS, Sham+MSC, CLP+ NS, and CLP + MSC groups (Figure 3(c)).

### 3.4. MSCs Transplantation Alleviated Microglial Activation of the Hippocampus of Sepsis Surviving Mice

**Figure 4:**
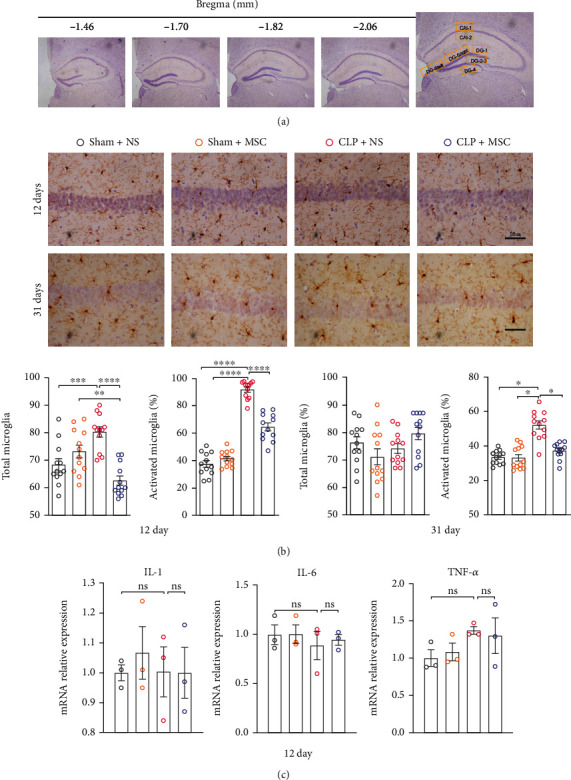
MSCs transplantation alleviated microglial activation of the hippocampus of sepsis surviving mice: (a) schematic diagram of four hippocampal levels and 7 counting areas in each hippocampal level; (b) representative images of Iba-1 staining (yellow), the number of total microglial cells, and the percentage of Iba-1^+^ activated microglia in the hippocampus on the 12^th^ and 31^th^ days after CLP surgery (Bar = 50 *μ*m, unpaired *t* test, *N* = 12 sections from 3 mice per group); (c) represent qPCR analysis of mRNA levels of inflammatory factors IL-1, IL-6, and TNF-*α* in the hippocampus on the 12^th^ day after CLP surgery (one-way ANOVA followed by multiple comparison tests, *N* = 3 per group). The data were expressed as mean ± SEM; ^∗^*P* < 0 · 05, ^∗∗^*P* < 0 · 01, ^∗∗∗^*P* < 0 · 001; Sham+NS: mice received laparotomy and tail vein injection of normal saline; Sham+MSC: mice received laparotomy and tail vein injection of MSCs; CLP + NS: mice received CLP surgery and tail vein injection of normal saline; CLP + MSC: mice received CLP surgery and tail vein injection of MSCs.

Neuroinflammation plays an important role in pathogenesis of post-sepsis cognitive dysfunction [11]. Thus, we detected microglial activation and inflammatory factor levels (IL-1, IL-6 and TNF-*α*) in the hippocampus. The percentages of activated microglial cells in CLP + NS group on the 12^th^ and 31^th^ days after CLP surgery significantly increased, relative to the Sham+NS, Sham+MSC, and CLP + MSC groups (Figures 4(a) and 4(b)). However, we did not observe differences in the mRNA levels of IL-1, IL-6, and TNF-*α* expressed in the the hippocampus among each group on the 12^th^ day after CLP surgery (Figure 4(c).

### 3.5. MSCs Transplantation Did Not Affect Peripheral Inflammation Level of Sepsis Surviving Mice

**Figure 5:**
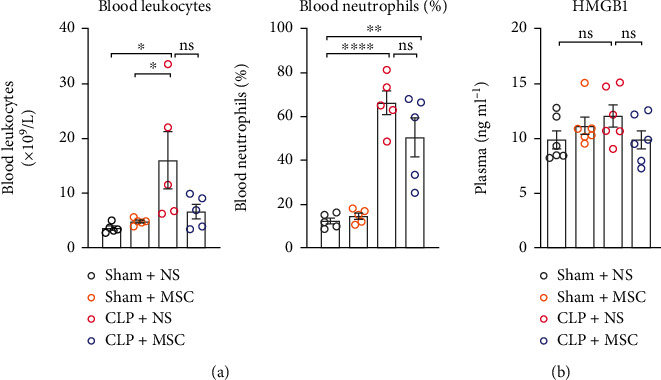
MSCs transplantation did not affect peripheral inflammation level of sepsis surviving mice. The leukocyte number and neutrophil percentages in peripheral blood on the 12^th^ day after CLP surgery (one-way ANOVA, *N* = 5 per group); (b) The HMGB1 protein level in peripheral blood on the 12^th^ day after CLP surgery (one-way ANOVA followed by multiple comparison tests, *N* = 6 per group). The data were expressed as the mean ± SEM; ^∗^*P* < 0 · 05, ^∗∗^*P* < 0 · 01, ^∗∗∗^*P* < 0 · 001. Sham+NS: mice received laparotomy and tail vein injection of normal saline; Sham+MSC: mice received laparotomy and tail vein injection of MSCs; CLP + NS: mice received CLP surgery and tail vein injection of normal saline; CLP + MSC: mice received CLP surgery and tail vein injection of MSCs.

Compared to the Sham+NS, Sham+MSC groups, the leukocyte amount and the neutrophil percentage in the peripheral blood of CLP + NS group significantly increased on the 12^th^ day after surgery, but the HMGB1 level did not (Figure 5(a) and 5(b)). There were no significant differences between CLP + NS group and CLP + MSC group in the leukocyte amount, the neutrophil percentage and the HMGB1 level of the peripheral blood on the 12^th^ day after surgery (Figure 5(a) and (b)).

## 4. Discussion

Post-sepsis cognitive impairment as a common post-sepsis sequela is influentially associated with discount life quality and decreased life independence in sepsis survivors [4, 5]. Our study tried to explore whether MSCs transplantation via tail vein at the later phase of sepsis could improve the post-sepsis cognitive function in sepsis surviving mice. We found that sepsis mice with MSCs transplantation at the later phase of sepsis showed better long-term memory and working memory in novel object recognition test and delayed matching-to-place (DMP) water maze task, respectively. No obvious side effects marked by weight and survival rate were detected. This was in line with previous study about MSCs transplantation at the early phase of sepsis [9]. They found that MSCs transplantation at the first 6 hours after sepsis was also protective for the cognitive function of septic surviving mice. These data may suggest that MSCs transplantation via peripheral vein is a simple, safe, and effective intervention for the prevention and treatment of post-sepsis cognitive impairment, although its impact on long-term outcomes or survival warrants further study.

Neuroinflammation plays an important role in pathogenesis of post-sepsis cognitive dysfunction [10, 22, 23]. Limiting neuroinflammation and reverting microcirculatory dysfunction improved post-sepsis cognitive function by the statins in experimental models, although they had been unprofitable in a randomized controlled clinic trial [24, 25]. In the study reported by Silva et al. [9], MSCs transplantation at the first 6 hours after sepsis improved post-sepsis cognitive function, corresponding to the inhibition of neuroinflammation and peripheral inflammation (marked by the levels of IL-1*β*, IL-6, and MCP1 in plasma) in mice. Thus, in this study, we also evaluated the peripheral inflammation with leukocyte number, neutrophil percentages, and HMGb1 level (a critical molecule in pathogenesis of sepsis [26]) in peripheral blood, and the neuroinflammation with microglial activation and inflammatory factors (IL-1,IL-6, and TNF-*α*) in the hippocampus. However, we found, MSCs transplantation at the later phase of sepsis had no significant effects on the peripheral inflammation and the level of hippocampal inflammatory factors, suggesting that anti-inflammation was not the underlying protective mechanism of MSCs transplantation at the later phase of sepsis. Interestingly, we found that MSCs transplantation at the later phase of sepsis significantly improved the neurogenesis impairment of septic surviving mice. These suggest that the protective mechanism of transplanted MSCs to post-sepsis cognitive function is sepsis-phase dependent. In addition, we did not trace transplanted MSCs in brain, but traced them in peripheral organs. Previous studies had shown that MSCs transplanted in the hippocampus could increase hippocampal neurogenesis in immunodeficient mice [27, 28]. Transplanted MSCs could promote tissue recovery and regeneration via secreting lots of specific mediators, conferring immunomodulatory, anti-inflammatory, antimicrobial, angiogenic, antifibrotic, and structural reparative properties [9, 18]. Notably, Islam et al. revealed that MSCs protected against acute lung injury via transferring its mitochondria to pulmonary alveoli [29]. This provides a further explanation for the protective mechanism of peripherally transplanted MSCs to central nervous system connected cognitive function in our study. However, the intermediators need further investigation.

## 5. Conclusion

**Figure 6:**
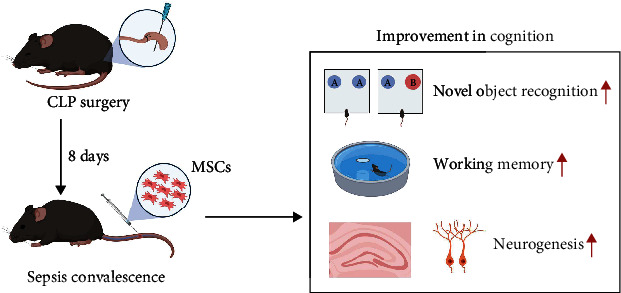
The graphical abstract of this study. MSCs transplantation can improve post-sepsis cognitive impairment and hippocampal neurogenesis of sepsis surviving mice via peripheral vein on the eighth day after CLP surgery.

In conclusion, our results demonstrated that MSCs transplantation via peripheral vein at later phase of sepsis could be considered as a ‘proof of concept' for the prevention and treatment of post-sepsis cognitive impairment (Figure 6). The effectiveness of this therapy and the underlying mechanism needed to be evaluated further in the future trial(s) and model animals.

## Figures and Tables

**Table 1:** Primers used for quantitative real-time PCR.

Target gene	Primers	Sequence(5′-3′)
GAPDH	Forward	CATGGCCTTCCGTGTTCCTA
	Reverse	TACTTGGCAGGTTTCTCCAGG
IL-1	Forward	CGCAGCAGCACATCAACAAGAGC
	Reverse	TGTCCTCATCCTGGAAGGTCCACG
IL-6	Forward	GCTTAATTACACATGTTCTCTGGGAAA
	Reverse	CAAGTGCATCGTTGTTCATAC
TNF-*α*	Forward	CCACCACGCTCTTCTGTCTA
	Reverse	GAGGCCATTTGGGAACTTCTCATC

## Data Availability

The data used to support the findings of this study are available from the corresponding author upon request.

## Supplementary Materials

Supplementary Materials Figure 1: MSC transplantation improved the hippocampal neurogenesis of sepsis survival mice. (a) Representative images of DAPI and doublecortin (DCX) staining in the hippocampus on the 12th 397 398 days after CLP surgery (bar = 50 *μ*m). (b) Representative images of doublecortin (DCX) staining in the hippocampus on the 12th 399 days after CLP surgery (bar = 20 *μ*m). (c) Representative images of DAPI and doublecortin (DCX) staining in the hippocampus on the 31th 400 days after CLP surgery (bar = 50 *μ*m). (d) Representative images of doublecortin (DCX) staining in the hippocampus on the 31th 401 days after CLP 402 surgery (bar = 20 *μ*m).

## References

[1] Shankar-Hari M., Phillips G. S., Levy M. L. (2016). Developing a new definition and assessing new clinical criteria for septic shock. *Journal of the American Medical Association*.

[2] Gofton T. E., Young G. B. (2012). Sepsis-associated encephalopathy. *Nature Reviews Neurology*.

[3] Widmann C. N., Heneka M. T. (2014). Long-term cerebral consequences of sepsis. *Lancet Neurology*.

[4] Iwashyna T. J., Ely E. W., Smith D. M., Langa K. M. (2010). Long-term cognitive impairment and functional disability among survivors of severe sepsis. *JAMA*.

[5] Iwashyna T. J., Cooke C. R., Wunsch H., Kahn J. M. (2012). Population burden of long-term survivorship after severe sepsis in older Americans. *Journal of the American Geriatrics Society*.

[6] Okeke E. B., Uzonna J. E. (2016). In search of a cure for sepsis: taming the monster in critical care medicine. *Journal of Innate Immunity*.

[7] Song C. G., Zhang Y. Z., Wu H. N. (2018). Stem cells: a promising candidate to treat neurological disorders. *Neural Regeneration Research*.

[8] Tajiri N., Duncan K., Antoine A. (2014). Stem cell-paved biobridge facilitates neural repair in traumatic brain injury. *Frontiers in Systems Neuroscience*.

[9] Silva A. Y. O., Amorim É. A., Barbosa-Silva M. C. (2020). Mesenchymal stromal cells protect the blood-brain barrier, reduce astrogliosis, and prevent cognitive and behavioral alterations in surviving septic mice. *Critical Care Medicine*.

[10] Golzari S. E., Mahmoodpoor A. (2014). Sepsis-associated encephalopathy versus sepsis-induced encephalopathy. *The Lancet Neurology*.

[11] Gazdic M., Volarevic V., Arsenijevic N., Stojkovic M. (2015). Mesenchymal stem cells: a friend or foe in immune-mediated diseases. *Stem Cell Reviews and Reports*.

[12] Rittirsch D., Huber-Lang M. S., Flierl M. A., Ward P. A. (2009). Immunodesign of experimental sepsis by cecal ligation and puncture. *Nature Protocols*.

[13] Wang X., Song Y., Chen J. (2020). Subcutaneous administration of *β*-hydroxybutyrate improves learning and memory of sepsis surviving mice. *Neurotherapeutics*.

[14] Ezzat T., Dhar D. K., Malago M., Olde Damink S. W. (2012). Dynamic tracking of stem cells in an acute liver failure model. *World Journal of Gastroenterology*.

[15] Qing W., Li F., Wang X., Quan C., Ouyang W., Liao Q. (2018). Inhibiting RIP1 improves chronic stress-induced cognitive impairments in D-galactose-induced aging mice. *Frontiers in behavioral neuroscience*.

[16] de Hoz L., Moser E. I., Morris R. G. (2005). Spatial learning with unilateral and bilateral hippocampal networks. *European Journal of Neuroscience*.

[17] Bizon J. L., LaSarge C. L., Montgomery K. S., McDermott A. N., Setlow B., Griffith W. H. (2009). Spatial reference and working memory across the lifespan of male Fischer 344 rats. *Neurobiology of Aging*.

[18] Chen J., Liu S., Wang X. (2022). HDAC6 inhibition alleviates anesthesia and surgery-induced less medial prefrontal dorsal hippocampus connectivity and cognitive impairment in aged rats. *Molecular Neurobiology*.

[19] Zhang S., Wang X., Ai S., Ouyang W., Le Y., Tong J. (2017). Sepsis-induced selective loss of NMDA receptors modulates hippocampal neuropathology in surviving septic mice. *PLoS One*.

[20] Krzisch M., Sultan S., Sandell J., Demeter K., Vutskits L., Toni N. (2013). Propofol anesthesia impairs the maturation and survival of adult-born hippocampal neurons. *Anesthesiology*.

[21] Wang C., Chen T., Li G., Zhou L., Sha S., Chen L. (2015). Simvastatin prevents *β*-amyloid_25-35_-impaired neurogenesis in hippocampal dentate gyrus through *α*7nAChR-dependent cascading PI3K-Akt and increasing BDNF *via* reduction of farnesyl pyrophosphate. *Neuropharmacology*.

[22] Michels M., Vieira A. S., Vuolo F. (2015). The role of microglia activation in the development of sepsis-induced long- term cognitive impairment. *Brain Behavior and Immunity*.

[23] Mazeraud A., Righy C., Bouchereau E., Benghanem S., Bozza F. A., Sharshar T. (2020). Septic-associated encephalopathy: a comprehensive review. *Neurotherapeutics*.

[24] Needham D. M., Colantuoni E., Dinglas V. D. (2016). Rosuvastatin versus placebo for delirium in intensive care and subsequent cognitive impairment in patients with sepsis-associated acute respiratory distress syndrome: an ancillary study to a randomised controlled trial. *The Lancet Respiratory Medicine*.

[25] Reis P. A., Alexandre P. C., D’Avila J. C. (2017). Statins prevent cognitive impairment after sepsis by reverting neuroinflammation, and microcirculatory/endothelial dysfunction. *Brain Behavior and Immunity*.

[26] Deng M., Tang Y., Li W. (2018). The endotoxin delivery protein HMGB1 mediates Caspase-11-dependent lethality in sepsis. *Immunity*.

[27] Munoz J. R., Stoutenger B. R., Robinson A. P., Spees J. L., Prockop D. J. (2005). Human stem/progenitor cells from bone marrow promote neurogenesis of endogenous neural stem cells in the hippocampus of mice. *Proceedings of the National Academy of Sciences of the United States of America*.

[28] Park D., Yang G., Bae D. K. (2013). Human adipose tissue-derived mesenchymal stem cells improve cognitive function and physical activity in ageing mice. *Journal of Neuroscience Research*.

[29] Islam M. N., Das S. R., Emin M. T. (2012). Mitochondrial transfer from bone-marrow-derived stromal cells to pulmonary alveoli protects against acute lung injury. *Nature medicine*.

