# Supplementary material for: Peripheral Transplantation of Mesenchymal Stem Cells at Sepsis Convalescence Improves Cognitive Function of Sepsis Surviving Mice
Source: Oxid Med Cell Longev. 2022 Sep 19;2022:6897765. doi: 10.1155/2022/6897765 (PMC9526624; doi:10.1155/2022/6897765)
Supplement: Supplementary Materials — Figure 1: MSC transplantation improved the hippocampal neurogenesis of sepsis survival mice. (a) Representative images of DAPI and doublecortin (DCX) staining in the hippocampus on the 12th 397 398 days after CLP surgery (bar = 50 μm). (b) Representative images of doublecortin (DCX) staining in the hippocampus on the 12th 399 days after CLP surgery (bar = 20 μm). (c) Representative images of DAPI and doublecortin (DCX) staining in the hippocampus on the 31th 400 days after CLP surgery (bar = 50 μm). (d) Representative images of doublecortin (DCX) staining in the hippocampus on the 31th 401 days after CLP 402 surgery (bar = 20 μm). [file 6897765.f1.docx]

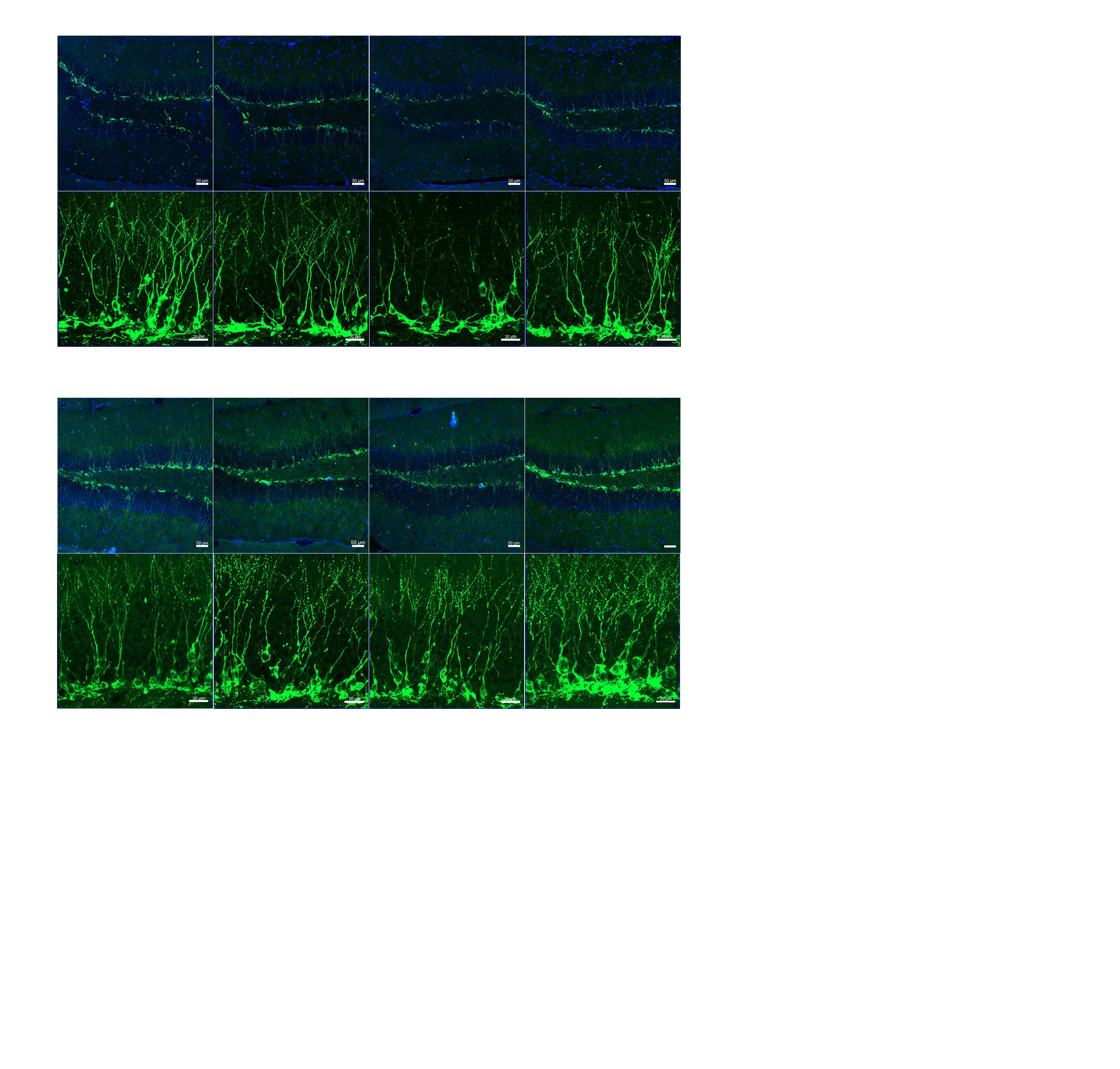

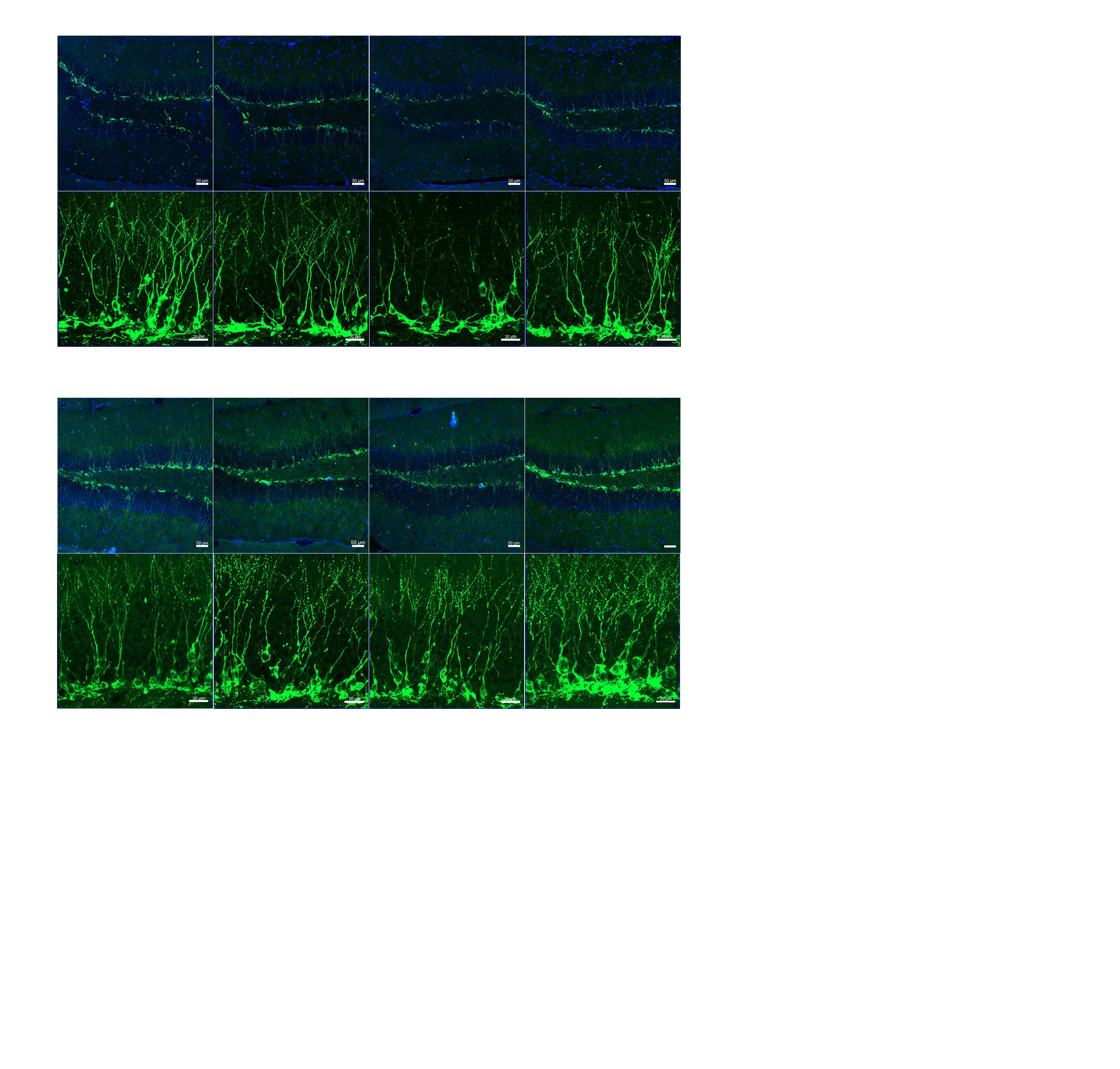


12day

31day

**→**

**MLo**

**MLm**

**GCL**

**MLi**

**SGZ**

**→**

**→**

**MLo**

**MLm**

**GCL**

**MLi**

**SGZ**

**→**

Sham+NS

Sham+MSC

CLP+NS

CLP+MSC

DCX+DAPI(X10)

DCX+DAPI(X10)

A

B

C

D

**Supplementary figure 1** MSCs transplantation improved hippocampal neurogenesis of sepsis survival mice (A) Representative images of DAPI and Doublecortin (DCX) staining in hippocampus on the 12th days after CLP surgery (bar=50μm). (B) Representative images of Doublecortin (DCX) staining in hippocampus on the 12th days after CLP surgery (bar=20μm). (C) Representative images of DAPI and Doublecortin (DCX) staining in hippocampus on the 31th days after CLP surgery (bar=50μm). (D) Representative images of Doublecortin (DCX) staining in hippocampus on the 31th days after CLP surgery (bar=20μm).
